# Supplementary material for: Association between Cardiovascular Risk and Diabetes with Colorectal Neoplasia: A Site-Specific Analysis
Source: J Clin Med. 2018 Nov 27;7(12):484. doi: 10.3390/jcm7120484 (PMC6306807; doi:10.3390/jcm7120484)
Supplement: Supplementary file 1 [file jcm-07-00484-s001.pdf]

Supp Table 1. Characteristics of Subjects with CAD history without angiographically verified CAD and with CAD history with angiographically verified CAD

Variable	CAD history	Angiographically verified CAD	P-value†
N	53	55	
Age (years, (mean±SD))	67.8 ± 7.3	65.5 ± 8.0	0.120
Males (n, (%))	29 (63.9)	40 (72.7)	0.051
BMI (kg/m^2)	28.0 ± 4.2	28.3 ± 4.8	0.754
Smoking status (never/ever/current (n, (%))	25 (47.2)/20 (37.7)/8 (15.1)	24 (43.6)/26 (47.4)/5 (9)	0.482
Hypertension (n, (%))	34 (64.2)	36 (65.5)	0.887
Type 2 diabetes mellitus (n, (%))	11 (20.7)	16 (29.1)	0.301
Metabolic Syndrome (n, (%))	20 (37.7)	19 (34.5)	0.730
FRS	11.3 ± 7.2	11.8 ± 6.5	0.737
HS	5.3 ± 4.3	4.3 ± 3.6	0.192
Aspirin (n, (%))	36 (76.9)	47 (67)	0.031

†for this comparison, subjects with CAD history but without coronary angiography (n=53) were compared to angiographically verified CAD (n=55)

In subjects with an angiographically verified CAD, OR for the detection of any adenoma and advanced neoplasia were 1.468 (0.835-2.583, p=0.182) and 3.126 (1.299-7.526, p=0.011). Characterisics of subjects with CAD history but without coronary angiography and subjects with angiographically verified CAD did not differ except for Apirin use. Framingham Risk Score (FRS); Heart Score of the European Society of Cardiology (HS)

Previously, the Framingham risk score (FRS) was used to distinguish low, intermediate and high cardiovascular risk by using 0-10%, 11-20% and >20% (FRS 10-20) as cut-off values. Likewise, the cut-off values of Heart Score of the European Society of Cardiology (HS) were <1%, 1-5%, >5% (HS 1-5) or 0-3%, 3-6% und>6% (HS 3-6). Because of statistical reasons we did not follow these suggestions; however, as supplementary data (Supplementary Tables 2-5) we report our results with these strata. [62, 63]

Supp Table 2. Risk of any adenoma or advanced neoplasia by FRS and HS risk strata

		Any lesion					Advanced neoplasia			
	Total number of subjects	Subjects with any adenoma	Number of subjects to be screened	OR (95% CI)	p		Subjects with advanced neoplasia	Number of subjects to be screened	OR (95% CI)	p
FRS (10-20)	1990	526 (26.4%)	3.8				75 (3.8)	26.5		
Low	1344	279 (20.8%)	4.8	Reference			32 (2.4)	42.0	Reference	
Intermediate	600	228 (38%)	2.6	2.34 (1.89-2.89	<0.001		40 (6.7)	15.0	2.93 (1.82-4.71)	<0.001
High	46	19 (41.3%)	2.4	2.69 (1.47-4.90)	0.001		3 (6.5)	15.3	2.86 (0.84-9.71)	0.092
HS (1-5)	1990	526 (26.4%)	3.8				75 (3.8)	26.5		
Low	635	91 (14.3%)	7.0	Reference			10 (1.6)	63.5		
Intermediate	900	290 (29.3%)	3.4	2.48 (1.92-3.22)	0.000		38 (3.8)	26.1	2.49 (1.23-5.04)	0.011
High	365	145 (39.7%)	2.5	3.94 (2.90-5.35)	<0.001		27 (7.4)	13.5	4.99 (2.39-10.44)	<0.001
HS (3-6)	1990	526 (26.4%)	3.8				75 (3.8)	26.5		
Low	1464	325 (22.2%)	4.5	Reference			40 (2.6)	36.6		
Intermediate	160	98 (61.3%)	1.6	2.16 (1.63-2.85)	<0.001		18 (11.3)	8.9	2.67 (1.51-4.76)	0.001
High	160	103 (64.4%)	1.6	2.27 (1.71-2.99)	<0.001		17 (10.6)	9.4	2.47 (1.38-4.42)	0.002

Suppl Table 3. Associations of Framingham Score Strata with colorectal lesion by location

		Any adenoma			
FRS (10-20)	Total number of subjects	Subjects with any adenoma	Number of subjects to be screened	OR (95% CI)	p
	1990	526	3.8		
Proximal Colon					
Low	1344	169 (12.6%)	8.0	Reference	
Intermediate	600	146 (24.3%)	4.0	2.24 (1.75-2.86)	0.000
High	46	10 (21.7%)	4.6	1.93 (0.94-3.96)	0.073
Distal Colon					
Low	1344	121 (9.0%)	11.1	Reference	
Intermediate	600	114 (19.0%)	5.3	2.37 (1.80-3.13)	0.000
High	46	12 (26.1%)	3.8	3.57 (1.80-7.07)	0.000
Rectum					
Low	1344	46 (3.4%)	29.2	Reference	
Intermediate	600	44 (7.3%)	13.6	2.23 (1.46-3.42)	0.000
High	46	3 (6.5%)	15.3	1.97 (0.59-6.58)	0.271

Suppl Table 4. Associations of Heart Score Strata <1%, 1-5%, >5% with colorectal lesion by location

		Any adenoma			
HS (1-5)	Total number of subjects	Subjects with any adenoma	Number of subjects to be screened	OR (95% CI)	p
	1990	526	3.8		
Proximal Colon					
Low	635	51 (8.0%)	12.5	Reference	
Intermediate	990	180 (18.2%)	5.5.	2.55 (1.83-3.54)	0.000
High	365	94 (25.8%)	3.9	3.97 (2.74-5.75)	0.000
Distal Colon					
Low	635	38 (6.0%)	16.7	Reference	
Intermediate	990	136 (13.7%)	7.3	2.50 (1.72-3.64)	0.000
High	365	73 (20%)	5.0	3.93 (2.59-5.97)	0.000
Rectum					
Low	635	18 (2.8%)	35.3	Reference	
Intermediate	990	52 (5.3%)	19.0	1.90 (1.10-3.28)	0.021
High	365	23 (6.3%)	15.9	2.31 (1.23-4.33)	0.009

Suppl Table 5. Associations of Heart Score Strata 0-3%, 3-6% und>6%. with colorectal lesion by location

		Any adenoma			
HS (3-6)	Total number of subjects	Subjects with any adenoma	Number of subjects to be screened	OR (95% CI)	p
	1990	526	3.8		
Proximal Colon					
Low	1464	192 (13.1%)	7.6	Reference	
Intermediate	160	67 (41.9%)	2.3	2.33 (1.70-3.20)	0.000
High	160	66 (41.3%)	2.4	2.23 (1.6-3.06)	0.000
Distal Colon					
Low	1464	155 (10.7%)	9,4	Reference	
Intermediate	160	41 (25.6%)	3.9	1.60 (1.10-2.33)	0.013
High	160	51 (31.9%)	3.1	2.04 (1.44-2.89)	0.000
Rectum					
Low	1464	61 (4.2%)	24	Reference	
Intermediate	160	20 (12.5%)	8	1.94 (1.15-3.27)	0.013
High	160	12 (9.3%)	13.3	1.10 (0.59-2.08)	0.760
